# Predicting human disease mutations and identifying drug targets from mouse gene knockout phenotyping campaigns

**DOI:** 10.1242/dmm.038224

**Published:** 2019-05-07

**Authors:** Robert Brommage, David R. Powell, Peter Vogel

**Affiliations:** 1 Department of Metabolism Research, Lexicon Pharmaceuticals, 8800 Technology Forest Place, The Woodlands, TX 77381, USA; 2 St. Jude Children's Research Hospital, Pathology, MS 250, Room C5036A, 262 Danny Thomas Place, Memphis, TN 38105, USA

**Keywords:** Knockout mice, Mouse models, Phenotyping, Phenomics, Translational medicine

## Abstract

Two large-scale mouse gene knockout phenotyping campaigns have provided extensive data on the functions of thousands of mammalian genes. The ongoing International Mouse Phenotyping Consortium (IMPC), with the goal of examining all ∼20,000 mouse genes, has examined 5115 genes since 2011, and phenotypic data from several analyses are available on the IMPC website (www.mousephenotype.org). Mutant mice having at least one human genetic disease-associated phenotype are available for 185 IMPC genes. Lexicon Pharmaceuticals' Genome5000™ campaign performed similar analyses between 2000 and the end of 2008 focusing on the druggable genome, including enzymes, receptors, transporters, channels and secreted proteins. Mutants (4654 genes, with 3762 viable adult homozygous lines) with therapeutically interesting phenotypes were studied extensively. Importantly, phenotypes for 29 Lexicon mouse gene knockouts were published prior to observations of similar phenotypes resulting from homologous mutations in human genetic disorders. Knockout mouse phenotypes for an additional 30 genes mimicked previously published human genetic disorders. Several of these models have helped develop effective treatments for human diseases. For example, studying *Tph1* knockout mice (lacking peripheral serotonin) aided the development of telotristat ethyl, an approved treatment for carcinoid syndrome. *Sglt1* (also known as *Slc5a1*) and *Sglt2* (also known as *Slc5a2*) knockout mice were employed to develop sotagliflozin, a dual SGLT1/SGLT2 inhibitor having success in clinical trials for diabetes. Clinical trials evaluating inhibitors of AAK1 (neuropathic pain) and SGLT1 (diabetes) are underway. The research community can take advantage of these unbiased analyses of gene function in mice, including the minimally studied ‘ignorome’ genes.

## Abstract (precis)

**Summary:** Large-scale, focused phenotyping campaigns provide data for thousands of mutant mouse genes, yielding key information for understanding rare human diseases and for developing novel drug therapies.

## Introduction

Understanding gene function can explain the disease phenotypes observed in carriers of common genetic variants and deleterious mutations. Great progress is being made, deciphering the functions of the ∼20,000 human genes, but the actions of many genes remain poorly understood. For example, the Undiagnosed Diseases Network and other DNA sequencing efforts can typically identify gene mutations for one-third of patients with unknown rare genetic diseases (Splinter et al., 2018). The genes, and their actions, responsible for the remaining patients remain unknown. Identifying the actions and biochemical pathways of disease genes provides insights for potential therapies. Although imperfect, mice are the best-established models for human disease (Justice and Dhillon, 2016; Perlman, 2016; Sundberg and Schofield, 2018; Nadeau and Auwerx, 2019). This article summarizes data from two large-scale mouse gene knockout phenotyping campaigns: the International Mouse Phenotyping Consortium (IMPC) and Lexicon Pharmaceuticals' Genome5000™ program.

Both campaigns employed reverse genetics, the approach that relies on analyzing the phenotypes that result from the inactivation of specific genes to provide information on the physiological functions of these genes, to generate knockout mouse strains. Forward genetics approaches, involving the identification of the genes responsible for mouse phenotypes resulting from spontaneous mutations (Davisson et al., 2012) or chemical mutagenesis (Probst and Justice, 2010; Arnold et al., 2012; Sabrautzki et al., 2012; Potter et al., 2016; Wang et al., 2018), have also made major contributions to our understanding of genetic disease. Besides identifying inactivating gene mutations, forward genetics approaches often identify hypomorphic, gain-of-function and dominant-negative mutations. For example, The Jackson Laboratory (JAX) employed whole-exome sequencing to decipher spontaneous pathogenic mutations in 124 mouse strains (Fairfield et al., 2015; Palmer et al., 2016).

## Mouse gene knockout phenotyping

Although examining mutant mice in individual laboratories has uncovered the functions of many genes, such piecemeal studies have several limitations. First, individual research groups often focus on the systems in which they have interest, hypotheses and experimental expertise. As a result, they can miss or ignore additional phenotypes. For example, a behavior laboratory can easily overlook concurrent immune disorders. Second, since research groups tend to individualize experimental techniques, comparisons among different laboratories can be difficult. Mouse strains, sex and age, along with assays and computational analyses, also vary. Third, there is a strong bias in the community to repeatedly study well-characterized genes, leaving thousands of genes, known as the ‘ignorome’ or the ‘dark genome’, unexplored (Edwards et al., 2011; Pandey et al., 2014; Oprea et al., 2018; Stoeger et al., 2018). The Mouse Genome Informatics database (Eppig, 2017) includes 13,924 genes with published mutant alleles in mice (data correct as of 19 February 2019), indicating that 6000 mouse genes remain unexplored and are therefore part of the ignorome.

Large-scale mutant mouse phenotyping campaigns that employ a panel of assays covering a wide range of phenotypes and apply standardized experimental protocols and statistical analyses can address these limitations. The thousands of genes examined in these projects include both ignorome and previously characterized genes. Technical staff generally have no knowledge of purported gene functions, which minimizes subconscious bias, and the large amounts of data collected from wild-type mice allow tracking of possible variations from normal values over time (Moore et al., 2018a).

Two large-scale mouse gene knockout phenotyping campaigns have been undertaken: Lexicon Pharmaceuticals' Genome5000™ campaign, designed to identify novel drug targets, and the International Mouse Phenotyping Consortium (IMPC), which aims to characterize mutant phenotypes for all ∼20,000 mammalian genes. As summarized in Table 1, these two campaigns have many similarities but also differences.

**Table 1.**
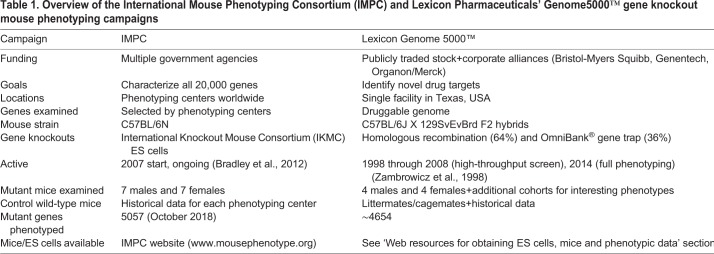
**Overview of the International Mouse Phenotyping Consortium (IMPC) and Lexicon Pharmaceuticals' Genome5000™ gene knockout mouse phenotyping campaigns**

Lexicon's high-throughput phenotyping analyses were performed between 2001 and the end of 2008, and included alliances with Bristol-Myers Squibb (Toyn et al., 2010; Kostich et al., 2016), Genentech (Tang et al., 2010) and Organon/Merck. The ongoing IMPC effort evolved from and includes data for 449 genes obtained during earlier European Mouse Disease Clinic (EUMODIC) and Sanger Mouse Genetics Program (MGP) mutant mouse phenotyping campaigns (Ayadi et al., 2012; White et al., 2013; de Angelis et al., 2015). Individual IMPC phenotyping centers select the genes they examine based on institutional investigator interests. Two focused mouse gene knockout phenotyping campaigns included examinations of 36 genes coding for glycan-binding proteins and glycosyltransferases (Orr et al., 2013) and 54 testes-expressed genes for male fertility (Miyata et al., 2016). In the early 2000s, Deltagen generated 750 mouse gene knockout lines using standard homologous recombination techniques (Moore, 2005) and phenotypic data are publicly available for 134 of these knockout lines (Table S1).

The IMPC effort utilizes murine embryonic stem (ES) cells generated by the International Knockout Mouse Consortium (IKMC) (Bradley et al., 2012; Brown et al., 2018). The IMPC phenotyping screen generally examines seven male and seven female mutant mice, with comparisons to phenotyping center-specific male and female historical control wild-type mice, which are shared among all genes examined (Meehan et al., 2017; Brown et al., 2018). An example of IMPC control data for body bone mineral density (BMD) is provided in Fig. S1. Lexicon's effort utilized ES cells generated by gene-trap mutagenesis using the OmniBank^®^ I library (Abuin et al., 2007; Hansen et al., 2008) or homologous recombination involving a λ-phage knockout shuttle system (Wattler et al., 1999). Phenotypes of *Adipor1*, *Angptl4*, *Ptprg*, *Rpn13* (also known as *Adrm1*) and *Tph1* mouse knockout lines generated independently via both ES cell technologies were identical. The Lexicon primary phenotyping screen generally examined four male and four female mutant mice, with comparisons to both littermate/cagemate and historical control wild-type mice. The parents of the mutant mice examined initially were subsequently mated a second time to provide a second cohort of mice for possible replication studies. The primary screen clearly identified dramatic phenotypes (*Alpk3*, *Brs3*, *Ksr2*, *Lrrk1*, *Mc4r* and *Sost*), with milder phenotypes confirmed or refuted with the second cohort. This approach follows the Bayesian statistical paradigm. If phenotypic replication was successful and the gene encoded a potential drug target, multiple additional cohorts of mutant mice were generated for sophisticated analyses. For example, more than 700 homozygous mutant mice were generated for *Aak1*, *Dagla*, *Ksr2*, *Ptprg*, *Sglt1* (also known as *Slc5a1*), *Sglt2* (also known as *Slc5a2*), *Stk4* and *Tph1* genes.

Both Lexicon and the IMPC employ similar phenotyping screens for audiology, behavior, blood cell counts, cardiology, body BMD and composition, immunology, metabolism, ophthalmology, radiology and serum chemistry. When gene knockout was lethal, yielding no adult homozygous mice, both campaigns examined mutant heterozygous mice. Beyond the common screening assays discussed above, Lexicon examined cortical and trabecular bone architecture by micro computed tomography (microCT) (Brommage et al., 2014), pain sensitivity by hot plate and formalin skin responses (Kostich et al., 2016), neuronal amyloid-β levels (Toyn et al., 2010) and comprehensive histopathology (Schofield et al., 2012). Metabolic responses to feeding a high-fat diet were analyzed in a second cohort (Brommage et al., 2008). Whereas IMPC extends the embryonic lethal analysis to time of death and high-throughput optical projection and microCT imaging (Dickinson et al., 2016), Lexicon did not examine the developmental abnormalities responsible for embryonic lethality.

The IMPC publishes detailed mutant mouse phenotype data. These publications include histopathology for 50 genes (Adissu et al., 2014); plasma metabolic profiling for 62 genes (Probert et al., 2015); skin, hair and nail abnormalities for 169 genes (Sundberg et al., 2017); developmental abnormalities for 401 embryonic-lethal knockout lines (Dickinson et al., 2016); skin data from 500+ genes (DiTommaso et al., 2014; Liakath-Ali et al., 2014); whole-mount LacZ reporter tissue expression profiles (Armit, 2015) in adult mice for 313 (West et al., 2015) and 424 (Tuck et al., 2015) genes; hearing data for 3006 genes (Bowl et al., 2017); metabolic phenotyping for 2016 genes (Rozman et al., 2018); and ophthalmic data for 4364 genes (Moore et al., 2018b). A manuscript summarizing IMPC bone data and relationships to human skeletal diseases is in preparation. The IMPC website (www.mousephenotype.org) provides comprehensive mutant mouse phenotype data in a readily searchable format (Koscielny et al., 2014). Updates of ongoing progress in IMPC mouse phenotyping continue, with Release 9.2 (5614 phenotyped genes) published in January 2019.

All high-throughput screens have false positives and false negatives (Karp et al., 2010) and ‘…it has never been easier to generate high-impact false positives than in the genomic era’ (MacArthur, 2012). The occurrence of false negatives can be estimated by the ability to identify the expected phenotypes arising from knockouts of benchmark genes, which are associated with well-established human and mouse mutant phenotypes. Examples of successful benchmark gene confirmation include *Brs3*, *Cnr1* and *Mcr4* in Lexicon's obesity screen (Brommage et al., 2008), and *Crtap*, *Lrp5*, *Ostm1*, *Src* and *Sost* in Lexicon's bone screen (Brommage et al., 2014). Conversely, researchers can detect false positives by phenotyping additional cohorts of mutant mice. The IMPC campaign provides data for the primary screen only, and statistical modeling calculations (Karp et al., 2010) estimate an 11.4% false-positive rate averaged among all IMPC phenotyping assays. Lexicon's primary screen included fewer mice than that of the IMPC, and many false positives, subsequently identified with secondary screens, were observed.

Complete and variably penetrant lethality are common in gene knockout mice (Wilson et al., 2017). The IMPC defines subviable mutant lines as having fewer (<12.5% of the litter) than the expected 25% surviving homozygous mice resulting from heterozygous crosses (http://www.mousephenotype.org/data/embryo). The latest IMPC data for 4969 mutant lines show 24% preweaning lethality and 10% subviability. Lexicon observed ∼16% preweaning lethality among 4654 mutant lines (Brommage et al., 2014).

Two IMPC phenotyping centers (Freudenthal et al., 2016; Dyment et al., 2016; Rowe et al., 2018) perform specialized skeletal analyses beyond the body BMD and radiology data obtained as part of the high-throughput screen (Table S1). Combined bone quantitative X-ray microradiography (Butterfield et al., 2019) and bone breaking strength data are available for 100 genes, with skeletal phenotypes observed for nine genes (Bassett et al., 2012). Gene knockout of the murine *Slc20a2* phosphate transporter (Beck-Cormier et al., 2019) results in skeletal defects and brain calcification, mimicking the homologous human genetic disease. Integration of IMPC mouse bone data and human genome-wide association study (GWAS) of heel bone BMD and fracture data from the UK Biobank identified variants in *GPC6* (Kemp et al., 2017) and *DAAM2* (Morris et al., 2019) as key determinants of skeletal health.

A summary of Lexicon's phenotyping campaign (∼4654 genes, with 3762 viable adult homozygous gene knockout lines undergoing bone phenotyping) was published in 2014 (Brommage et al., 2014). Published phenotypes involving multiple cohorts of knockout mice are available for 100 genes summarized below.

## Modeling human Mendelian genetic disorders

Mutant mice contribute to our understanding of the mechanisms responsible for human genetic disorders. The IMPC performs an automated comparison of mutant mouse phenotypes to over 7000 rare human diseases in the Online Mendelian Inheritance in Man (OMIM) and Orphanet databases. The comprehensive 2017 update (Meehan et al., 2017) summarizes IMPC disease model discovery findings. Briefly, of the 3328 IMPC mouse genes examined, 621 had previous MGI mouse model annotations, with 385 genes (62%) having common observed phenotypes. Importantly, 90% (8984 of 9942) of the gene-phenotype annotations described by the IMPC had not previously been described in the literature. From the OMIM or Orphanet databases, 889 known rare disease-gene associations have an orthologous IMPC mouse mutant displaying at least one phenotype. These 889 associations involve 185 IMPC genes for which mutant mice showed at least one human disease-associated phenotype. Details on these data are available in supplementary tables 1-4 in Meehan et al. (2017). Updates to these analyses are provided within the ‘Human Diseases’ section of the IMPC website.

**Fig. 1.**
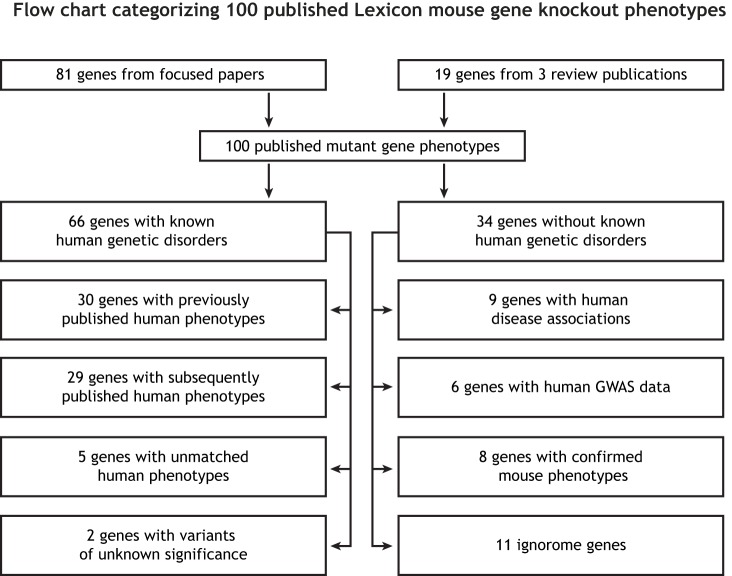
**Flow chart categorizing 100 published Lexicon mouse gene knockout phenotypes.** We grouped these based on known or unknown human-mouse gene associations.

Lexicon published mouse knockout phenotypes for 100 genes (Fig. 1; Table S2) in both focused papers (*N*=81) and summaries (*N*=19) on obesity (Brommage et al., 2008) and bone phenotypes (Brommage et al., 2014), and the Genentech Secreted Protein Discovery Initiative (SPDI) gene alliance (Tang et al., 2010). Manual annotation of the PubMed database (www.ncbi.nlm.nih.gov/pubmed) identified human Mendelian disease phenotypes for 66 of these 100 mouse genes, with the remaining 34 having no known associated human Mendelian genetic disorder. Table 2 lists 30 genes for which Lexicon's mutant mouse data support previously identified human phenotypes. All 30 genes have an OMIM disease designation.

**Table 2.**
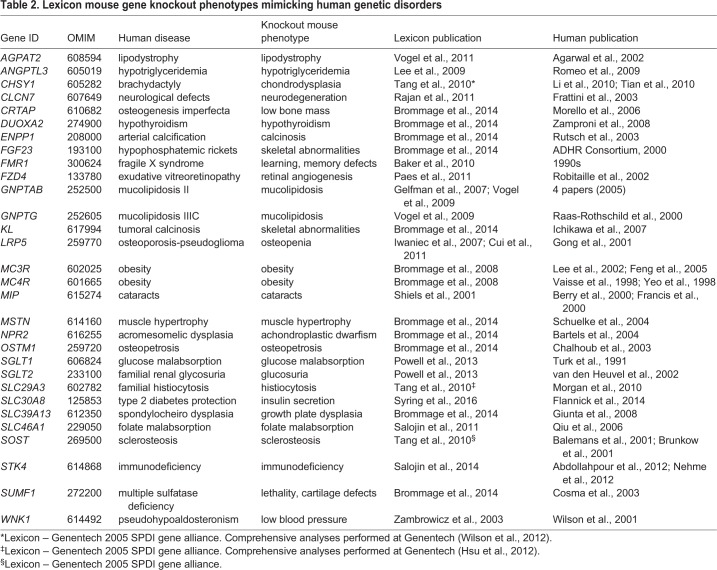
**Lexicon mouse gene knockout phenotypes mimicking human genetic disorders**

Importantly, 29 mutant mouse phenotypes mimicking human disease phenotypes were characterized and published prior to the identification of their orthologous human disease genes (Table 3). Eighteen of these 29 genes have an OMIM disease designation, and OMIM summaries for many of the remaining 11 genes are outdated. At the time of Lexicon's mouse phenotypic analyses, most of these 29 genes were minimally studied ignorome genes.

**Table 3.**
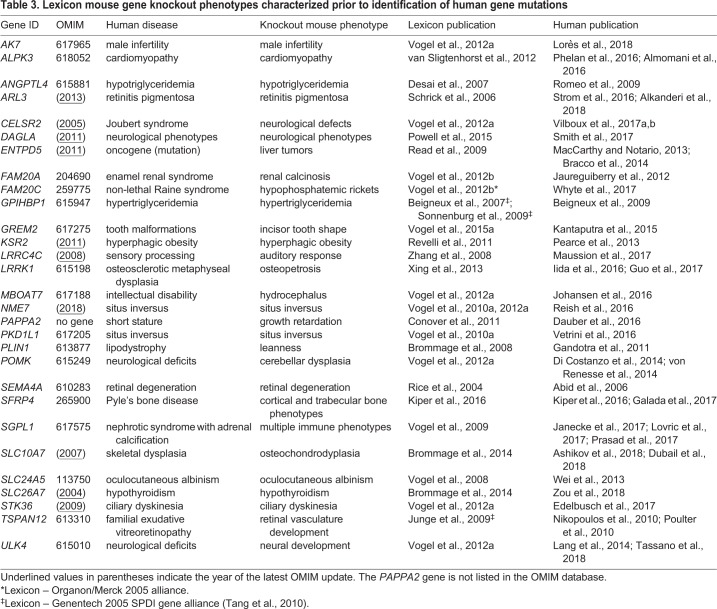
**Lexicon mouse gene knockout phenotypes characterized prior to identification of human gene mutations**

*ADIPOR1* (Rice et al., 2015) and *HDAC4* (Rajan et al., 2009) are classified as variants of unknown significance in OMIM, as subsequent human studies did not confirm the initial disease phenotype-gene associations observed in humans (Zhang et al., 2016) and mice. *Hdac4* knockout mice are presently in the IMPC phenotyping queue. *Adipor1* mice showed abnormal retinal morphology in both Lexicon and IMPC screens. Diverging human and mouse phenotypes have been described for five genes [*PTPRG* (Zhang et al., 2012), *RRM2B* (Powell et al., 2005), and *SLC25A1*, *SLC30A5* and *SLC30A10* (Brommage et al., 2014)], which can result from incomplete human and/or mouse phenotypic evaluations (Table 4). For example, human *SLC30A5* mutations affecting a zinc transporter reduce human breast milk zinc content without other clinical observations (Kumar et al., 2015), whereas *Slc30a5* mutant mice have low bone mass (Inoue et al., 2002; Brommage et al., 2014), but mouse milk composition was not examined. In the IMPC campaign, *Slc30a10* mice are currently in the phenotyping queue, *Slc25a1* mice exhibited preweaning lethality and the other three genes have not been examined yet.

**Table 4.**
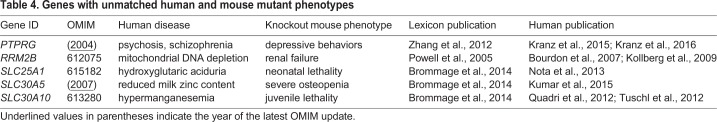
**Genes with unmatched human and mouse mutant phenotypes**

The 34 Lexicon mouse phenotypes described without corresponding published human Mendelian genetic disorders fall into several categories (Table 5). Mouse mutant phenotypes for *Slc6a4* [selective serotonin reuptake inhibitor (SSRI) drug target] and *Tph1* (carcinoid syndrome drug target) have been examined by independent laboratories, but not by the IMPC. Human GWAS data exist for *EPHA6*, *FADS1*, *KCNK16*, *NOTUM*, *TPH2* and *WNT16*, with the IMPC having examined *Epha6*, *Fads1*, *Notum* and *Wnt16* mutant mice and observing preweaning lethality in *Notum* and *Wnt16* knockout mice. Multiple studies indicate that *ATG4B*, *CLDN18*, *LIMK2*, *MDM4*, *MKP1* (also known as DUSP1), *RPN13* and *UCHL5* are human oncogenes, with the IMPC having examined *Atg4b*, *Cldn18*, *Limk2*, *Mkp1* and *Uchl5* mutant mice and observing preweaning lethality in *Limk2* and *Uchl5* knockout animals. There is minimal published information for 11 ignorome genes (*Aak1*, *Ak8*, *Dpcd*, *Itfg2*, *Kif27*, *Kirrel1*, *Nme5*, *Tmem218*, *Tmub1*, *Tomm5* and *Ttll1*), and the IMPC examined only preweaning lethal *Ttll1* mutant mice*.* Independently published mouse knockout data exist for eight genes (*Brs3*, *Fam20b*, *Pik3c2a*, *Rock1*, *Rock2*, *Sh2d3c*, *Slc30a7* and *Spns2*), with the IMPC having examined *Pik3c2a*, *Rock1* and *Spns2* mutant mice, observing preweaning lethality in *Pik3c2a* and *Rock1* mutant mice.

**Table 5.**
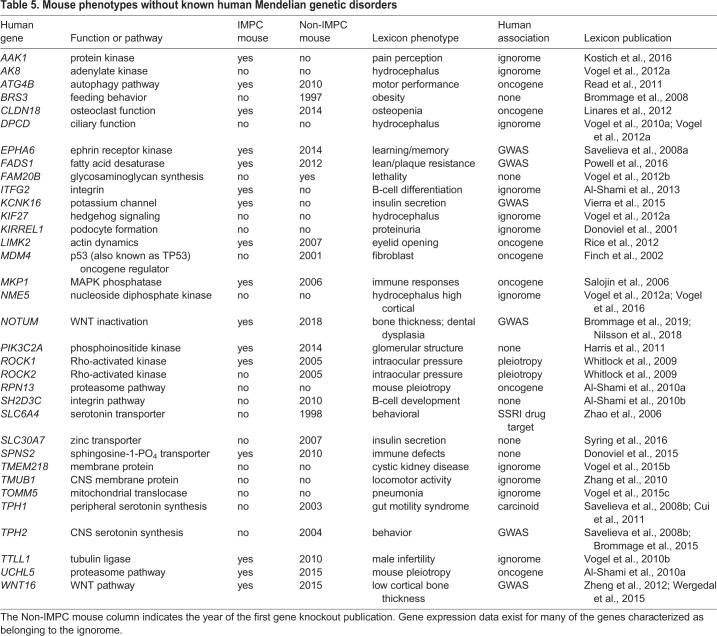
**Mouse phenotypes without known human Mendelian genetic disorders**

Of the 100 Lexicon genes summarized in Fig. 1, embryonic lethality was observed in *Fam20b*, *Mdmx* and *Wnk1* knockout mice, perinatal lethality in *Kirrel1* knockout mice, and juvenile lethality in *Arl3*, *Clcn7*, *Fgf23*, *Klotho*, *Npr2*, *Ostm1*, *Slc4a1* and *Sumf1* knockout mice. Subviability, defined as a deviation from the expected 1-2-1 Mendelian ratio of wild-type, heterozygous and homozygous mice from heterozygous crosses at *P*<0.001 by Chi-squared testing, was observed in *Angptl4* (754, 17%), *Notum* (931, 19%), *Pkd1l1* (61, 10%), *Pomk* (395, 10%), *Rock1* (197, 15%), *Rock2* (227, 4%), *Rpn13* (39, 9%) and *Uchl5* (644, 14%) mice. Numbers in parentheses indicate observed numbers of wild-type mice and percentages of homozygous mutant mice, respectively.

Studies in mutant mice can also provide guidance for treating human genetic diseases. For example, Lexicon (Iwaniec et al., 2007) and others (Sawakami et al., 2006) showed that teriparatide treatment increases bone mass in *Lrp5* gene knockout mice with low bone mass. Similarly, teriparatide treatment increased BMD in a patient with osteoporosis pseudoglioma syndrome resulting from an inactivating *LRP5* mutation (Arantes et al., 2011).

## IMPC – Lexicon comparisons

These two successful phenotyping campaigns had different objectives, funding and approaches to phenotypic screening (Table 1), and comprehensive comparisons are beyond the scope of this article. Of the 100 Lexicon genes discussed here, 36 were also examined by the IMPC. Preweaning lethality was observed for 15 genes (*Fam20c*, *Fzd4*, *Limk2*, *Mboat7*, *Notum*, *Pik3c2a*, *Rock1*, *Sgpl1*, *Slc25a1*, *Slc46a1*, *Stk36*, *Sumf1*, *Ttll1*, *Uchl5* and *Wnt16*) in the IMPC, but not the Lexicon, phenotypic analyses. The OMIM autosomal-recessive disease genes *FAM20C*, *MBOAT7*, *SGPL1*, *SLC46A1* and *SUMF1* are not expected to exhibit disease phenotypes in the heterozygous mutant mice examined by the IMPC. Lexicon examined F2 hybrid C56BL/6J X 129SvEv-Brd mice, and hybrid vigor presumably contributed to better viability compared to the purebred C57BL/6N mice examined by the IMPC. The lower rate of lethality across all genes examined (∼16% for Lexicon versus 25% for IMPC) is consistent with this hypothesis. Incomplete penetrance is common in human inherited diseases (Cooper et al., 2013) and variations in modifier genes likely contribute to this variable penetrance (Riordan and Nadeau, 2017).

Of the 36 genes examined in both phenotyping campaigns, 17 genes model human Mendelian disease. Both campaigns provided robust mouse data consistent with human genetic disorders involving mutations of *ALPK3*, *ANGPT4*, *DAGLA*, *DUOXA2*, *LRRK1* and *SLC24A5*. *Lrrk1* mice have the highest body volumetric BMD and BMD values in the Lexicon and IMPC screens, respectively (Fig. S1). Preweaning lethality and/or subviability of homozygous mice in the IMPC screen for *Fam20c*, *Fzd4*, *Grem2*, *Mboat7*, *Sgpl1*, *Slc25a1*, *Slc46a1* and *Sumf1* precluded the evaluation of homozygous knockout phenotypes for these genes*.* In contrast to observations by Lexicon, the IMPC did not observe soft tissue calcification in *Fam20a* mice, situs inversus in *Nme7* mice, nor any phenotypes in *Sglt2* (only immunological parameters were examined) or *Slc30a8* mice. Human gene mutation phenotypes for *FAM20A* (enamel renal syndrome), *NME7* (situs inversus), *SGLT2* (familial renal glycosuria) and *SLC30A8* (resistance to Type 2 diabetes) are consistent with Lexicon's mutant mouse phenotypes.

Lexicon's extended phenotyping allowed characterization of human disease phenotypes not measured in the initial high-throughput screening assays. For example, human *DUOXA2* (Zamproni et al., 2008) and *SLC26A7* (Zou et al., 2018) mutations result in hypothyroidism, and Lexicon observed abnormal thyroid gland histology in both gene knockouts. Moreover, Lexicon's *Slc26a7* mice had reduced circulating thyroxine levels (Brommage et al., 2014).

## Identifying novel drug targets

Studying human genetic disorders (Plenge et al., 2013; Nelson et al., 2015; Williams, 2016) in conjunction with knockout mice (Zambrowicz and Sands, 2003) can identify previously unknown tractable targets and lead to effective drugs. *PCSK9* is an example of this strategy, as knowledge that human inactivating mutations result in hypocholesterolemia led to the development of neutralizing antibodies to treat this condition (Jaworski et al., 2017). *Pcsk9* knockout mice are also hypocholesterolemic (Rashid et al., 2005) and blood cholesterol levels are halved in IMPC mice.

Unlike the genome-wide effort of the IMPC, Lexicon's choice of genes for knockout mouse analyses emphasized the druggable genome (Plewczynski and Rychlewski, 2009; Finan et al., 2017; Santos et al., 2017), which includes enzymes, receptors, ligands, channels and secreted proteins. Ideally, drugs should influence disease processes without adversely affecting healthy tissues. In addition to identifying novel drug targets from beneficial mutant phenotypes, human genetic diseases and global gene knockout mice quickly identify or, preferably rule out, the possible adverse phenotypes that are likely to contribute to secondary drug target effects. For example, hypocholesterolemic subjects with inactivating *PCKS9* mutations and IMPC *Pcks9* mutant mice have no unexpected health problems related to this mutation, suggesting that therapeutic inhibition of PCKS9 activity should be safe. Generally, once this approach identifies novel drug targets, the preclinical drug development pipeline involves establishing robust enzymatic or binding assays, screening chemical libraries, optimization of chemical structures for potency and pharmacokinetic properties, followed by increasingly sophisticated animal pharmacology and toxicology studies. Thus, Lexicon stopped examining new mouse gene knockouts after December 2008 and stopped all basic research after January 2014 to focus on clinical development of small molecule drugs against selected targets previously identified in its gene knockout phenotyping campaign.

Lexicon's preclinical drug development program included the generation of neutralizing antibodies against ANGPTL3 (Lee et al., 2009), ANGPTL4 (Desai et al., 2007), DKK1 (Brommage et al., 2014), FZD4 (Paes et al., 2011) and NOTUM (Brommage et al., 2019). Treating wild-type mice with each antibody successfully replicated the phenotypes observed in knockout mice. Subsequent work by Regeneron Pharmaceuticals demonstrated the efficacy of anti-ANGPTL3 antibodies for the treatment of hypercholesterolemia in human patients (Dewey et al., 2017). In addition to providing phenotypic information, gene knockout mice provide two advantages in antibody generation and characterization. First, producing antibodies should, theoretically, be more efficient in specific gene knockout compared to wild-type mice, as the knockout mouse immune systems have never been exposed to the immunizing proteins. Second, lack of antibody specificity is a major experimental problem, and the ‘… most stringent control for antibody specificity requires comparison of antibody reactivity in wild-type tissues or cells to reactivity in knockout animals…’ (Schonbrunn, 2014). Lexicon demonstrated the specificities of its anti-ANGPTL3 and anti-ANGPTL4 antibodies by showing lack of reactivity to tissues from the corresponding gene knockout mice.

In addition to antibodies, Lexicon developed small-molecule chemical inhibitors to 12 targets and information on these targets is provided in Table S3. Orally active inhibitors of AAK1, SGLT1, SGLT2, SGPL1, SLC6A7 and TPH1 entered human clinical trials. Lexicon's peripheral serotonin synthesis inhibitors LX1031 and telotristat ethyl (both acting on tryptophan hydroxylase 1 encoded for by the *TPH1* gene) showed efficacy in subjects with irritable bowel syndrome (Brown et al., 2011) and carcinoid syndrome (Kulke et al., 2017), respectively. Telotristat ethyl was approved for the treatment of carcinoid syndrome in 2017. Neither drug crosses the blood-brain barrier to inhibit the neuronal TPH2 serotonin-synthesizing enzyme. Sotagliflozin, a dual SGLT1/SGLT2 glucose transport inhibitor, showed efficacy in Phase 3 trials for Type 1 diabetes (Garg et al., 2017) and in Phase 2 trials for Type 2 diabetes (Rosenstock et al., 2015), and is currently being developed, in collaboration with Sanofi, for both indications. Early clinical development is underway examining inhibitors of SGLT1 for Type 2 diabetes (Goodwin et al., 2017) and AAK1 for neuropathic pain (Kostich et al., 2016).

Although drug development is not formally part of its mission or funding, the IMPC generates important knowledge for drug target identification and precision medicine initiatives (Lloyd et al., 2015). Unlike Lexicon, the main goal of the IMPC is not drug development. However, we believe that its data and collaborative nature are an unmatched resource for future downstream work, both aimed at improving our fundamental understanding of mammalian gene function and at applying this knowledge to treatment of human genetic diseases.

## Conclusions

The IMPC and Lexicon mouse gene knockout phenotyping campaigns provide key data for scientists studying mouse and human genomics. By continually updating its online database, the IMPC increasingly characterizes ignorome genes. The future success of the IMPC in identifying gene functions of significance to human health can be expected based on the results of Lexicon's successful mutant mouse phenotyping efforts. Lexicon's clinical drug development efforts, aiming for approval of SGLT1, SGLT2 and AAK1 inhibitors, continue and their success should help patients with diabetes and neuropathic pain. We anticipate that future work will develop additional drugs from Lexicon's knowledge base and, with adequate support, that of the IMPC. Both campaigns are expected to continue to contribute key mouse data for researchers studying ignorome genes associated with human genetic diseases.

Although this article focuses on published results, we stress that networking and presenting preliminary mouse data at conferences facilitates interactions with scientists working in human genomics and can contribute to collaborations ultimately resulting in publication of newly identified human genetic data. Successful examples of this process include *FAM20A*, *GREM2* (Kantaputra et al., 2015), *KSR2* (Pearce et al., 2013) and *SFRP4* (Kiper et al., 2016), and GWAS data for *WNT16* (Medina-Gomez et al., 2012; Zheng et al., 2012; Wergedal et al., 2015). Lexicon collaborated with academic scientists on many projects, and IMPC collaborations with academia and pharma should be encouraged. Recent publications involving IMPC mouse bone data and human data from the UK Biobank (Kemp et al., 2017; Morris et al., 2019) should stimulate additional collaborations in the future.

We encourage scientists to visit the IMPC website for further understanding of the actions of genes of interest. As Francis Collins, Director of the US National Institutes of Health, stated in 2006, ‘A graduate student shouldn't spend a year making a knockout that's already been made. It's not a good use of resources’ (Grimm, 2006). IMPC data showing either lethality, lack of a specific phenotype of interest, presence of this phenotype, and/or presence of additional phenotypes can guide research decisions for individual laboratories and optimize the use of limited resources.

## Web resources for obtaining ES cells, mice and phenotypic data

The Mouse Genome Informatics (MGI) website (www.informatics.jax.org) is an excellent source of information on the availability of genetically modified mice. The IMPC website provides information on obtaining ES cells and cryopreserved sperm made available through the IKMC. The Monash University Embryonic Stem Cell (ES Cell)-to-Mouse Service group has published their experiences, from a ‘client’ perspective, using IKMC ES cells obtained worldwide (Cotton et al., 2015).

Information from individual IMPC phenotyping centers and the publicly available data from Deltagen and Lexicon are available in Table S1.

## Note added in proof

Sotagliflozin has been approved within the European Union for use as an adjunct to insulin therapy to improve glycemic control in adults with Type 1 diabetes and a body mass index ≥27 kg/m^2^, who could not achieve adequate glycemic control despite optimal insulin therapy.

## Supplementary Material

Supplementary information
