# Supplementary material for: Predicting human disease mutations and identifying drug targets from mouse gene knockout phenotyping campaigns
Source: Dis Model Mech. 2019 May 7;12(5):dmm038224. doi: 10.1242/dmm.038224 (PMC6550044; doi:10.1242/dmm.038224)
Supplement: Supplementary information [file dmm-12-038224-s1.pdf]

Figure S1. High Bone Mass in *Lrrk1* Gene Knockout Mice

Osteosclerotic metaphyseal dysplasia (OMIM 615198) results from human *LRRK1* mutations (Iida et al., 2016; Guo et al., 2017).

A: Histogram from Figure 1A of Lexicon publication showing *Lrrk1* mice have the highest body vBMD of all 3,629 viable gene knockout lines examined (Xing et al., 2013).

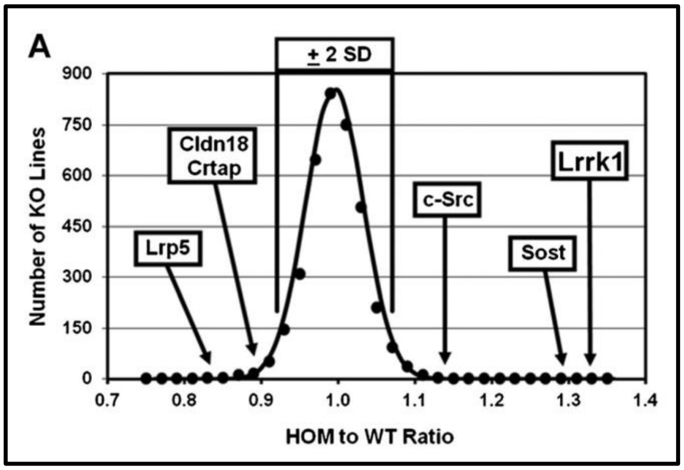

B: Histogram (left) and scatterplot (right) from IMPC website (November 2018). The histogram shows *Lrrk1* mice have the highest body BMD of all evaluated lines. Shown in orange bars are data from all mutant lines having an observed BMD phenotype. The scatterplot shows values for *Lrrk1* mice (blue) compared to all wild-type control mice examined at the MRC Harwell Phenotyping Center.

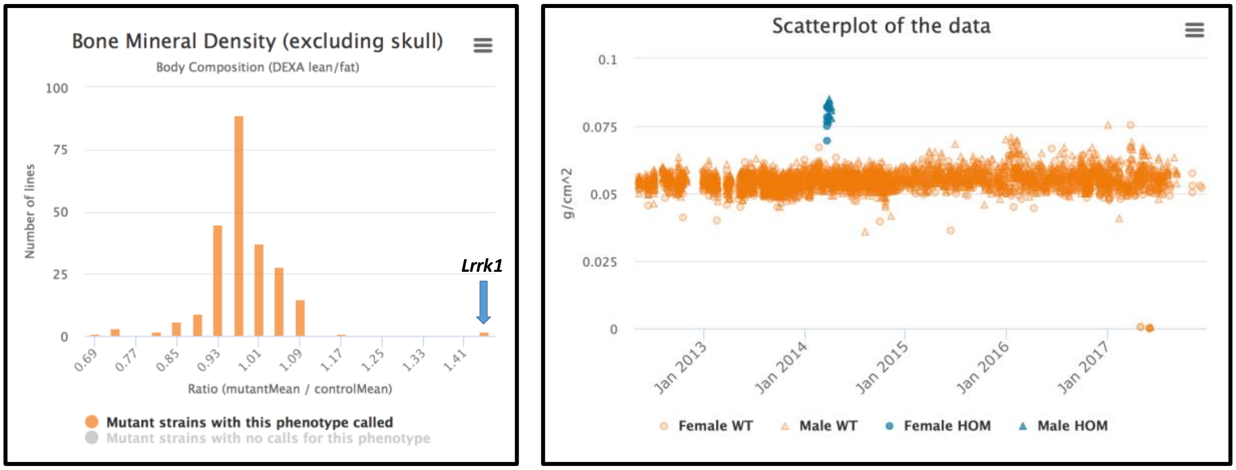

www.mousephenotype.org/data/phenotypes/MP:0010119

http://www.mousephenotype.org/data/charts?accession=MGI:2142227&allele_accession_id=MGI:5692778&zygosity=homozygote&parameter_stable_id=IMPC_DXA_004_001&pipeline_stable_id=HRWL_001&phenotyping_center=MRC%20Harwell

Disease Models & Mechanisms • Supplementary information

C: Whole-mount LacZ staining showing *Lrrk1* expression in adult ribs from a male mouse (53 days of age).

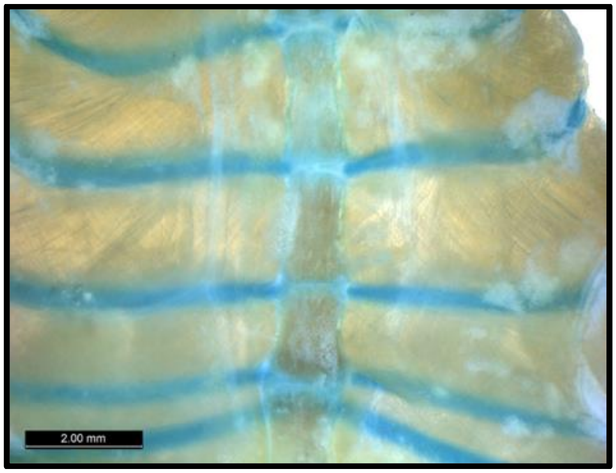

www.kompphenotype.org/lacz-tab.php?gene=Lrrk1&project=komp

Full experimental details are provided in the paper by West et al., 2015.

D: Unpublished Lexicon 4X images of sternum from adult wild-type (left) and *Lrrk1* gene knockout (right) mice showing osteosclerosis resulting from persistence of primary spongiosa adjacent to the growth plates and established trabeculae within the midshaft. Bones were obtained from mice at 79 weeks of age.

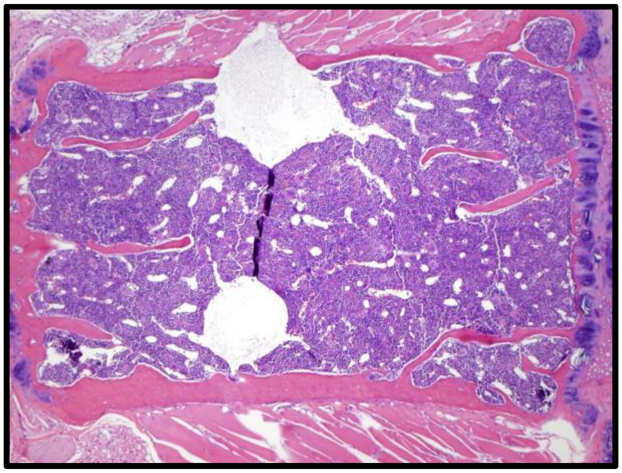

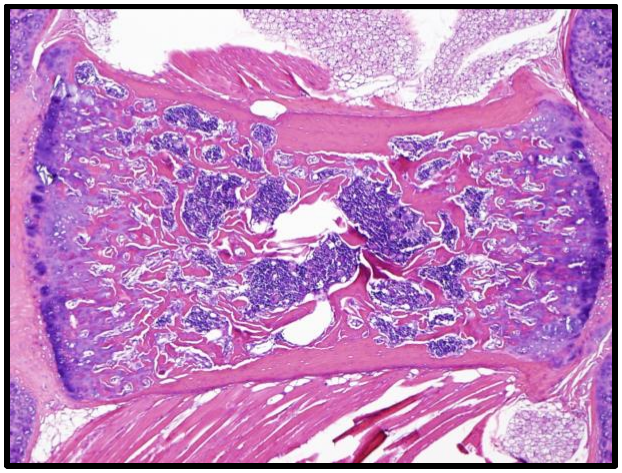

Legend: Lexicon and IMPC data for *Lrrk1* mutant mice.

Table S1. WEB RESOURCES FOR OBTAINING ES CELLS, MICE AND PHENOTYPIC DATA

MAIN IMPC PHENOTYPING CENTERS

In addition to the IMPC website, IMPC Phenotyping Centers have their own websites presenting data in various formats.

1) Australian Phenomics Facility at https://jcsmr.anu.edu.au/research/facilities/apf

2) Baylor College of Medicine (Houston) at www.bcm.edu/research/advanced-technology-core-labs/lab-listing/mouse-metabolic-and-phenotyping-core

3) Toronto Center for Phenogenomics at http://phenogenomics.ca/index2.html?v=1

4) German Mouse Clinic (Munich) at www.mouseclinic.de

5) Infrafrontier Mouse Disease Models at www.infrafrontier.eu

6) Institute Clinique de la Souris (Strasbourg) at www.ics-mci.fr/en/

7) JAX at www.jax.org/research-and-faculty/resources/mouse-mutant-resource/available-models

8) MRC Harwell at www.mousebook.org

9) Nanjing Biomedical Research Institute at www.scienceexchange.com/labs/nanjing-biomedical-research-institute-of-nanjing-university-nbri-nju

10) Sanger Mouse Genetics Program at www.sanger.ac.uk/science/collaboration/mouse-resource-portal

11) University of California-Davis KOMP Repository at www.komp.org

12) Korea Mouse Phenotyping Center at http://mousephenotype.kr

WEB RESOURES FOR FINDING ES CELLS and MUTANT MICE

International Mouse Strain Resource (Eppig et al., 2015) at www.findmice.org/repository

European Mouse Mutant Cell Repository (Schick et al., 2016) at https://www.eummcr.org

INFRAFRONTIER Resources (Raess et al., 2016) at https://www.infrafrontier.eu

DELTAGEN

Deltagen sperm and embryos (N = 216) listed at MMRRC.

http://www.findmice.org/summary?query=deltagen&states=Any&_states=1&types=Any&_types=1&repositories=Any&_repositories=1&_mutations=on#myDataTable=results%3D100%26startIndex%3D0%26sort%3Dscore%26dir%3D

Disease Models & Mechanisms • Supplementary information

Deltagen genes in MGI (N = 99)

http://www.informatics.jax.org/batch/summary#myDataTable=results%3D100%26startIndex%3D0

Deltagen phenotypes in MGI (N = 134)

http://www.informatics.jax.org/allele/reference/J:101679

LEXICON PHARMACEUTICALS

Lexicon ES cells, sperm, mice and gene knockout phenotype data can be obtained from multiple sources.

1) Lexicon contributed C57BL/6N gene trap knockout ES cells (Hansen et al., 2008), available through the Texas Institute for Genomic Medicine (TIGM), to the IKMC (Collins et al., 2007). These ES cells are distinct from the 129 SvEv Brd ES cells employed by Lexicon for its Genome 5000™ campaign. As of October 2018, studies of mutant mice obtained from TIGM ES cells resulted in 182 publications.

(www.tigm.org/publications/)

2) During 2005 the USA NIH provided funds for Lexicon to make available 126 gene knockout mouse lines

www.informatics.jax.org/knockout_mice/

and corresponding phenotypic data

www.informatics.jax.org/marker/reference/J:103485#myDataTable=results%3D100%26startIndex%3D100%26sort%3Ddefault%26dir%3Ddesc)

3) Gene knockout mice (N = 495) from the Genentech/Lexicon SPDI gene alliance examining trans-membrane and secreted proteins (Tang et al., 2010) are available from the MMRRC facility at the University of California-Davis

www.mmrrc.org/catalog/overview_Major_Collection.php

4) For 4000+ Lexicon genes, ES cells and/or cryopreserved sperm are available for purchase through the Taconic Biosciences Knockout Repository (www.taconic.org).

5) Lexicon ES cells, sperm and embryos (N = 3029) listed at MMRRC.

(http://www.findmice.org/summary?query=lexicon&states=Any&_states=1&types=Any&_types=1&repositories=Any&_repositories=1&_mutations=on#myDataTable=results%3D100%26startIndex%3D0%26sort%3Dscore%26dir%3D)

SPECIALIZED BONE ASSAYS

Two IMPC Phenotyping Centers perform specialized bone assays beyond the IMPC body BMD and dysmorphology screens and provide their data online.

Disease Models & Mechanisms • Supplementary information

1) The Molecular Endocrinology Laboratory at Imperial College London collaborates with WTSI (Welcome Trust Sanger Institute) and provides data for 620 mutant mouse lines as of November, 2018 (Freudenthal et al., 2016).

www.boneandcartilage.com/

2) Scientists at the University of Connecticut and the University of Rochester collaborate with JAX (Jackson Laboratories) and provide data for 220 mutant mouse lines as of November, 2018 (Dyment et al., 2016, Rowe et al., 2018).

http://bonebase.org/bonebase/

UC-DAVIS KOMP TISSUE LacZ EXPRESSION DATABASE

LacZ Expression Analysis in Whole-mount or Microscopic Tissue Sections (West et al., 2015).

www.kompphenotype.org/lacZmap.php

REFERENCES

Collins FS, Finnell RH, Rossant J, Wurst W. (2007). A new partner for the international knockout mouse consortium. Cell. 129, 235.

Eppig JT, Motenko H, Richardson JE, Richards-Smith B, Smith CL. (2015). The International Mouse Strain Resource (IMSR): Cataloging worldwide mouse and ES cell line resources. Mamm Genome. 26, 448-455.

Hansen GM, Markesich DC, Burnett MB, Zhu Q, Dionne KM, Richter LJ, Finnell RH, Sands AT, Zambrowicz BP, Abuin A. (2008). Large-scale gene trapping in C57BL/6N mouse embryonic stem cells. Genome Res. 18, 1670-1679. [Abstract/Full Text]

Raess M, de Castro AA, Gailus-Durner V, Fessele S, Hrabě de Angelis M; INFRAFRONTIER Consortium. (2016). INFRAFRONTIER: a European resource for studying the functional basis of human disease. Mamm Genome. 7-8, 445-450.

Schick JA, Seisenberger C, Beig J, Bürger A, Iyer V, Maier V, Perera S, Rosen B, Skarnes WC, Wurst W. (2016). CRISPR-Cas9 enables conditional mutagenesis of challenging loci. Sci Rep. 6, 32326.

Table S2. 100 Lexicon Genes with Published Mouse Knockout Phenotypes

A classification of Lexicon genes by comparison to homologous human genes.

66 Genes with Known Human Genetic Disorders

- **30 Mouse Genes with Previously Published Human Phenotypes**
  - ***Agpat2, Angptl3, Chsyl1, Clcn7, Crtap, Duoxa2, Enpp1, Fgf23, Fmr1, Fzd4, Gnptab, Gnptg, Kl, Lrp5, Mc3r, Mc4r, Mstn, Mip, Npr2, Ostm1, Sglt1, Sglt2, Slc29a3, Slc30a8, Slc39a13, Slc46a1, Sost, Stk4, Sumf1, Wnk1***
- **29 Mouse Genes Previewing Subsequently Published Human Phenotypes**
  - ***Ak7, Alpk3, Angptl4, Arl3, Celsr2, Dagla, Entpd5, Fam20a, Fam20c, Gpihbp1, Grem2, Krs2, Lrrc4c, Lrrk1, Mboat7, Nme7, Pappa2, Pkd1l1, Plin1, Pomk, Sem4a, Sfrp4, Sgpl1, Slc10a7, Slc24a5, Slc26a7, Stk36, Tspan12, Ulk4***
- **5 Mouse Genes with Unmatched Human - Mouse Mutant Phenotypes**
  - ***Ptprg, Rrm2b, Slc25a1, Slc30a5, Slc30a10***
- **2 Mouse Genes with Human Variants of Unknown Significance**
  - ***Adipor1, Hdac4***

34 Genes without Known Human Genetic Disorders

- **2 Mouse Genes with Human Drug Targets**
  - ***Slc6a4, Tph1***
- **7 Mouse Genes with Human Oncogenes**
  - ***Atg4b, Cldn18, Limk2, Mdm4, Mkp1, Rpn13, Uchl5***
- **6 Mouse Genes with Human GWAS Data**
  - ***Epha6, Fads1, Kcnk16, Notum, Tph2, Wnt16***
- **11 *Ignorome* Genes**
  - ***Aak1, Ak8, Dpcd, Itfg2, Kif27, Kirrel1, Nme5, Tmem218, Tmub1, Tomm5, Ttll1***
- **8 *Genes with Independent Mouse KO Publications***
  - ***Brs3, Fam20b, Pik3c2a, Rock1, Rock2, Sh2d3c, Slc30a7, Spns2***

Disease Models & Mechanisms • Supplementary information

Table S3. Lexicon Small Molecule Chemical Inhibitors

Gene	Protein	Function	Physiological Effect	Lexicon Publications
AAK1	**AP2 associated kinase 1**	**signaling pathways**	**neuropathic pain**	**Kostich et al., 2016**
DCK	**deoxycytidine kinase**	**nucleoside metabolism**	**drug metabolism**	**Jessop et al., 2009 Tarver et al., 2009 Yu et al., 2016**
FADS1	**fatty acid desaturase 1**	**fatty acid desaturase**	**lipid metabolism**	**Baugh et al., 2015**
GPR88	**G protein-coupled receptor 88**	**orphan GPCR**	**CNS dopaminergic signaling**	**Bi et al., 2015 Dzierba et al., 2015**
LIMK2	**LIM domain kinase 2**	**cytoskeletal organization**	**intraocular pressure**	**Harrison et al., 2009**
NOTUM	**notum, palmitoyl-protein carboxyesterase**	**WNT inactivation**	**cortical bone thickness**	**Han et al., 2016 Tarver et al., 2016**
ROCK1/2	**Rho kinases 1 + 2**	**cytoskeletal organization**	**intraocular pressure**	**Harrison et al., 2014**
SGPL1	**sphingosine-1-phosphate lyase 1**	**sphingosine-1-phosphate catabolism**	**lymphocyte migration**	**Bagdanoff et al., 2010**
***SLC5A1* (SGLT1)**	**solute carrier family 5 member 1**	**intestinal + renal glucose transport**	**glucose absorption**	**Goodwin et al., 2017 Powell et al., 2017**
***SLC5A2* (SGLT2)**	**solute carrier family 5 member 2**	**renal glucose transport**	**human diabetes therapy**	**Powell et al., 2014 Powell et al., 2015**
***SLC6A7* (PROT)**	**sodium-dependent proline transporter**	**neuronal proline uptake**	**cognition**	**Yu et al., 2009 Zipp et al., 2014**
TPH1	**tryptophan hydroxylase 1**	**peripheral serotonin synthesis**	**gut motility**	**Liu et al., 2008 Shi et al., 2008 Cianchetta et al. 2010**

Medicinal chemistry efforts for AAK1 and GPR88 inhibitors were performed in collaboration with Bristol-Myers Squibb.

Disease Models & Mechanisms • Supplementary information

REFERENCES

Bagdanoff JT, Donoviel MS, Nouraldeen A, Carlsen M, Jessop TC, Tarver J, Aleem S, Dong L, Zhang H, Boteju L, et al. (2010). Inhibition of sphingosine 1-phosphate lyase for the treatment of rheumatoid arthritis: discovery of (E)-1-(4-((1R,2S,3R)-1,2,3,4526 tetrahydroxybutyl)-1H-imidazol-2-yl)ethanone oxime (LX2931) and (1R,2S,3R)-1-(2527 (isoxazol-3- yl)-1H-imidazol-4-yl)butane-1,2,3,4-tetraol (LX2932). J Med Chem. 53, 8650-8662.

Baugh SD, Pabba PK, Barbosa J, Coulter E, Desai U, Gay JP, Gopinathan S, Han Q, Hari R, Kimball SD, et al. (2015). Design, synthesis, and in vivo activity of novel inhibitors of delta-5 desaturase for the treatment of metabolic syndrome. Bioorg Med Chem Lett. 25, 3836-3839.

Bi Y, Dzierba CD, Fink C, Garcia Y, Green M, Han J, Kwon S, Kumi G, Liang Z, Liu Y, et al. (2015). The discovery of potent agonists for GPR88, an orphan GPCR, for the potential treatment of CNS disorders. Bioorg Med Chem Lett. 25, 1443-1447.

Cianchetta G, Stouch T, Yu W, Shi ZC, Tari LW, Swanson RV, Hunter MJ, Hoffman ID, Liu Q. (2010). Mechanism of inhibition of novel tryptophan hydroxylase inhibitors revealed by co- crystal structures and kinetic analysis. Curr Chem Genomics. 4, 19-26.

Dzierba CD, Bi Y, Dasgupta B, Hartz RA, Ahuja V, Cianchetta G, Kumi G, Dong L, Aleem S, Fink C, et al. (2015). Design, synthesis, and evaluation of phenylglycinols and phenyl amines as agonists of GPR88. Bioorg Med Chem Lett. 25, 1448-1452.

Goodwin NC, Mabon R, Harrison BA, Shadoan MK, Almstead ZY, Xie Y, Healy J, Buhring LM, DaCosta CM, Bardenhagen J, et al. (2009). Novel L-xylose derivatives as selective sodium dependent glucose cotransporter 2 (SGLT2) inhibitors for the treatment of type 2 diabetes. J Med Chem. 52, 6201-6204.

Han Q, Pabba PK, Barbosa J, Mabon R, Healy JP, Gardyan MW, Terranova KM, Brommage R, Thompson AY, Schmidt JM, et al. (2016). 4H-Thieno[3,2-c]chromene based inhibitors of Notum Pectinacetylesterase. Bioorg Med Chem Lett. 26, 1184-1187.

Harrison BA, Whitlock NA, Voronkov MV, Almstead ZY, Gu KJ, Mabon R, Gardyan M, Hamman BD, Allen J, Gopinathan S, et al. (2009). Novel class of LIM-kinase 2 inhibitors for the treatment of ocular hypertension and associated glaucoma. J Med Chem. 52, 6515-6518.

Harrison BA, Almstead ZY, Burgoon H, Gardyan M, Goodwin NC, Healy J, Liu Y, Mabon R, Marinelli B, Samala L, et al. (2014). Discovery and development of LX7101, a dual LIM kinase and ROCK inhibitor for the treatment of glaucoma. ACS Med Chem Lett. 6, 84-88.

Jessop TC, Tarver JE, Carlsen M, Xu A, Healy JP, Heim-Riether A, Fu Q, Taylor JA, Augeri DJ, Shen M, et al. (2009). Lead optimization and structure-based design of potent and bioavailable deoxycytidine kinase inhibitors. Bioorg Med Chem Lett. 19, 6784-6787.

Liu Q, Yang Q, Sun W, Vogel P, Heydorn W, Yu XQ, Hu Z, Yu W, Jonas B, Pineda R, et al. (2008). Discovery and characterization of novel tryptophan hydroxylase inhibitors that selectively inhibit serotonin synthesis in the gastrointestinal tract. J Pharmacol Exp Ther. 325, 47-55.

**Powell DR, DaCosta CM, Smith M, Doree D, Harris A, Buhring L, Heydorn W, Nouraldeen A, Xiong W, Yalamanchili P, et al. (2014).
Effect of LX4211 on glucose homeostasis and body composition in preclinical models. J Pharmacol Exp Ther. 350, 232-242.**

Powell DR, Smith MG, Doree DD, Harris AL, Xiong WW, Mseeh F, Wilson A, Gopinathan S, Diaz D, Goodwin NC, et al (2015). LP-925219 maximizes urinary glucose excretion in mice by inhibiting both renal SGLT1 and SGLT2. Pharmacol Res Perspect. 23, e00129.

Powell DR, Smith MG, Doree DD, Harris AL, Greer J, DaCosta CM, Thompson A, Jeter-Jones S, Xiong W, Carson KG, et al. (2017). LX2761, a sodium/glucose cotransporter 1 inhibitor restricted to the intestine, improves glycemic control in mice. J Pharmacol Exp Ther. 362, 85-97.

Shi ZC, Devasagayaraj A, Gu K, Jin H, Marinelli B, Samala L, Scott S, Stouch T, Tunoori A, Wang Y, et al. (2008). Modulation of peripheral serotonin levels by novel tryptophan hydroxylase inhibitors for the potential treatment of functional gastrointestinal disorders. J Med Chem. 51, 3684-3687.

Tarver JE, Jessop TC, Carlsen M, Augeri DJ, Fu Q, Healy JP, Heim-Riether A, Xu A, Taylor JA, Shen M, et al. (2009). 5-Fluorocytosine derivatives as inhibitors of deoxycytidine kinase. Bioorg Med Chem Lett. 19, 6780-6783.

Disease Models & Mechanisms • Supplementary information

Tarver JE Jr, Pabba PK, Barbosa J, Han Q, Gardyan MW, Brommage R, Thompson AY, Schmidt JM, Wilson AGE, He W, et al. (2016). Stimulation of cortical bone formation with thienopyrimidine based inhibitors of Notum Pectinacetylesterase. Bioorg Med Chem Lett. 26, 1525-1528.

Yu XC, Zhang W, Oldham A, Buxton E, Patel S, Nghi N, Tran D, Lanthorn TH, Bomont C, Shi ZC, et al. (2009). Discovery and characterization of potent small molecule inhibitors of the high affinity proline transporter. Neurosci Lett. 451, 212-216.

Yu XC, Miranda M, Liu Z, Patel S, Nguyen N, Carson K, Liu Q, Swaffield JC. (2016). Novel potent inhibitors of deoxycytidine kinase identified and compared by multiple assays. J Biomol Screen. 15, 72-79.

Zipp GG, Barbosa J, Green MA, Terranova KM, Fink C, Yu XC, Nouraldeen A, Wilson A, Savelieva K, Lanthorn TH, et al. (2014). Novel inhibitors of the high-affinity L-proline transporter as potential therapeutic agents for the treatment of cognitive disorders. Bioorg Med Chem Lett. 24, 3886-3890.

Disease Models & Mechanisms • Supplementary information
